# Commencement Exercises of the Chicago Medical College

**Published:** 1875-04-01

**Authors:** 


### COMMENCEMENT EXERCISES OF THE CHICAGO MEDICAL COLLEGE, MEDICAL DEPARTMENT OF NORTHWESTERN UNIVERSITY.

THE public exercises connected with the Sixteenth Annual Commencement in this College, took place on Monday and Tuesday, the 15th and 16th of March, 1875. On Monday afternoon, the candidates for graduation, with the Faculty of the College, and any members of the profession who chose to attend, assembled in the College Hall, and devoted two hours to the reading of theses, and the public examination of the candidates on the topics discussed in their respective papers. The class then repaired to the amphitheatre of the Mercy Hospital, where they witnessed the performance of the operation for the removal of stone from the bladder, by Prof. E. Andrews.

The patient was a boy, aged only four years; but the calculus was of large size.

On Tuesday, the Commencement Exercises, proper, commenced in the College Hall, at 2½ P.M. Before the hour, the room was completely filled by an audience of gentlemen and ladies. A good band of music was in attendance, and at suitable intervals, added much to the enjoyment of the occasion. Prayer was offered by the Rev. Arthur Edwards; the certificates of examination for the under graduates were presented by the Secretary of the Faculty. The Professor of Practical Surgery also presented special certificates to those who had served as assistants in the hospital. The Dean of the Faculty awarded the prize for the best thesis, to W. H. H. Hutton; and for the second best, to Chauncey A. Kelsey.

C. H. Fowler, D.D., President of the Northwestern University, then conferred the Degree of Doctor of Medicine on forty-five candidates, who had been duly recommended by the Faculty, and accompanied the same by a brief, but very appropriate charge. This was responded to, in behalf of the class, by H. D. Hardacker, A.B., in a very happy manner.

Then followed the formal Valedictory Address, by Prof. W. E. Quine, on *Success.* This performance was one of more than ordinary merit, and was listened to with close attention, and much real profit. The collegiate year just closed, appears to have been one of gratifying prosperity. The whole number of students in the different classes was 140. The number of Degrees conferred was forty-five, namely; three ad eundem, two honorary, and forty in the regular course. In regard to the preliminary education of the graduates in the regular course, we were gratified to learn that seven were graduates of literary institutions; and all but two had received more or less mental training in the higher schools and colleges of the country. We still hope the time will come, when no young man will think of commencing the study of medicine until he has a thorough mental training, and a good knowledge of the sciences.

